# The perspectives of iranian physicians and patients towards patient decision aids: a qualitative study

**DOI:** 10.1186/1756-0500-6-379

**Published:** 2013-09-25

**Authors:** Hamideh Rashidian, Saharnaz Nedjat, Reza Majdzadeh, Jaleh Gholami, Leila Haghjou, Bahar Sadeghi Abdollahi, Fereydoun Davatchi, Arash Rashidian

**Affiliations:** 1 Department of Epidemiology and Biostatistics, School of Public Health, Kerman University of Medical Sciences, Kerman, Iran; 2 Department of Epidemiology and Biostatistics, School of Public Health, Tehran University of Medical Sciences, Tehran, Iran; 3 Knowledge Utilization Research Center (KURC), Tehran University of Medical Sciences, Tehran, Iran; 4 Rheumatology Research Center, Tehran University of Medical Sciences, Shariati Hospital, Tehran, Iran; 5 Department of Health Management and Economics, School of Public Health, Tehran University of Medical Sciences, Tehran, Iran

**Keywords:** Patient decision aid tool, Qualitative study, Iran

## Abstract

**Background:**

Patient preference is one of the main components of clinical decision making, therefore leading to the development of patient decision aids. The goal of this study was to describe physicians’ and patients’ viewpoints on the barriers and limitations of using patient decision aids in Iran, their proposed solutions, and, the benefits of using these tools.

**Methods:**

This qualitative study was conducted in 2011 in Iran by holding in-depth interviews with 14 physicians and 8 arthritis patient. Interviewees were selected through purposeful and maximum variation sampling. As an example, a patient decision aid on the treatment of knee arthritis was developed upon literature reviews and gathering expert opinion, and was presented at the time of interview. Thematic analysis was conducted to analyze the data by using the OpenCode software.

**Results:**

The results were summarized into three categories and ten codes. The extracted categories were the perceived benefits of using the tools, as well as the patient-related and physician-related barriers in using decision aids. The following barriers in using patient decision aids were identified in this study: lack of patients and physicians’ trainings in shared decision making, lack of specialist per capita, low treatment tariffs and lack of an exact evaluation system for patient participation in decision making.

**Conclusions:**

No doubt these barriers demand the health authorities’ special attention. Hence, despite patients and physicians’ inclination toward using patient decision aids, these problems have hindered the practical usage of these tools in Iran - as a developing country.

## Background

There is strong evidence for the potential outcomes of a treatment and its effectiveness or harm in only 17% of cases. For others, which include most decisions, there is no definite evidence about the balance between their benefits and harms. In these cases, choosing the right treatment according to the patients’ conditions is difficult. Therefore, they need to be involved in the decision making process. Based on scientific evidence, taking patients’ opinions into consideration can ease decision making in such conditions [1,2].

Involving patients in medical decisions requires their awareness of all existing treatments, their outcomes and the extent to which treatments would correspond to their own conditions. Different studies have shown that using patient decision aids (PDA) can facilitate this process. Patient value is one of the main principles of evidence-based medicine and clinical decision making. Therefore, there is increasing focus on designing PDAs. PDAs have been provided in different forms of media such as decision boards, video CDs, audio tapes, e-books, pamphlets, and group presentations. They have made the existing evidence on treatment benefits and harms easier for patients to understand [3-7]. This tool also helps the patient select a treatment to his/her own benefit, based on his/her own values [7].

It is well documented that PDAs increase patients’ involvement in the process of decision making [2,3,5,6,8-14]. In fact, it gives them a more realistic view about diseases and their probable harms and side effects, and increases their assurance in decision making [5,6,8,15,16]. Moreover, this tool offers patients an active and collaborative role, leading to more effective, appropriate, safe, responsive and high quality health care [2].

More than 300 decision aids have been registered in the Cochrane Registry System and Ottawa Hospital website, all of which are from high-income countries, such as: United States, Canada, United Kingdom, Australia, Saudi Arabia, Netherlands and Germany, respectively in order of frequency [17]. The development of this tool has not yet been the focus of attention in developing countries. However, in Iran developing guidelines as a part of evidence-based medicine has begun a long time ago and is ongoing [18]. Nevertheless, developing the PDA as a tool for considering patients’ values and shared decision making has not gained any attention yet.

Evaluating the possibility of implementing this tool in Iran can result in useful information about the practicability of developing this tool in clinical settings. The present study has explained physicians’ and patients’ opinions about using this decision aid tool in Iran.

## Methods

This qualitative study was conducted in 2011 in Tehran, Iran, by holding in-depth interviews with 14 physicians and 8 arthritis patients.

The physicians and patients were selected through purposeful and maximum variation sampling. Sampling continued up to data saturation. Physicians were selected from a diverse range of specialties that could use PDAs. Both male and female patients from different socio-economical levels were interviewed. They were selected by visiting private and public rheumatology and orthopedic clinics. The specialists were asked to give the contact information of patients who were candidates of elective surgery. The interviewer then chose 5 females and 3 males from both upper and lower socio-economic statuses. The latter was estimated by their residential addresses. Each interviewee was asked to specify the place and time of the interview. Before beginning each interview, a sample of the designed tool was presented to the patients. For instance, a PDA on knee joint replacement surgery in severe arthritis was selected and translated to Persian by two physicians fluent in English; ultimately one version was provided from two copies. Then, 4 rheumatologists and orthopedists were interviewed to evaluate the tool’s content validity. Meanwhile, the specialty-specific English versions were presented along with the Persian versions to physicians of various specialties. They were given some time to go through the English and Persian versions before the interview began.

The interviewer was guided by a few open questions to explore the physicians’ opinions on the compatibility of tools with Iranian culture, the applicability of this tool in Iran, the advantages and disadvantages of using this tool, as well as the changes considered necessary for making the tools compatible in Iran.

During the patient interviews, after the Persian PDA tool was explained and presented, questions concerning patients’ rights, general opinions about their advantages and disadvantages, appropriateness of content and construct of the tool, and recommendations for upgrading the tool were asked. All interviews were performed by the first and second authors who were familiar with qualitative research methods and had experience in conducting qualitative interviews. A note-taker was also present during the interviews, which were audio-taped upon the interviewees’ permission and subsequently transcribed verbatim.

Data management was done with OpenCode software. Inductive thematic analysis was conducted to analyze the data. The codes and categories were extracted from the text of the transcripts. Each interview’s text was assumed as a whole and the fundamental meanings or its general context was described in one or a couple of paragraphs. Primary codes and categories were determined, then after frequent analysis, repeated categories and codes were merged. Member check was conducted to ensure the trustworthiness of the findings with all physicians. It was also conducted with two patients whom it was felt necessary to confirm the analysis of the findings with. All the interviews held with physicians were analyzed by a second person. But only two patient interviews were analyzed by a second person. However, since the second person had been present in all the interviews, the final analysis results of the patient interviews were approved by the second person as well. Where reliability was concerned, the inter-rater agreement between the two analyzers was greater than 90% for all the interviews.

Ethical approval for the study was obtained from the Ethics Committee of Tehran University of Medical Sciences (TUMS) (project code: 88-03-74-9467), which is in line with the Declaration of Helsinki. In the same context, participants were informed of the goals and significance of the research and written informed consents were obtained. They were asked for permission to record their voices and were reassured that all gathered information would only be used for research purposes, and would not be given to anyone other than research team members. Finally, a gift was given to each participant as a token of appreciation.

## Results

The participating physicians’ specialties by number were as follows: 3 endocrinologists, 2 neurologists, 2 general surgeons, 2 oncologists, 1 ophthalmologist, 1 gastroenterologist, 1 dermatologist, 1 cardiologist, and 1 urologist (14 in all). The patient participants included 5 women and 3 men. Two of the men and 3 women were selected from public hospitals and were of lower socio-economic status (LSES). The remaining participants were of upper socio-economic status (USES).

Three categories and ten codes were extracted from the interviews (Table 1). Each of the categories and their codes have been explained and for each the exact phrases expressed by some participants have been quoted in Italic font. The type of participant has also been parenthesized at the end of the quote in abbreviated form; e.g. woman from upper socio-economic status has been represented by ‘W-USES’. And specialists have been represented by the first few letters of each specialty, e.g. ophthalmologist by ‘Ophth’ and endocrinologist by ‘Endo’.

**Table 1:** Codes and categories extracted from the interviews

**Category**	**Code**
**A: Realized benefits of using PDAs**	1. Enhancing the psychological impact of treatment
	2. Adherence to principles of medical ethics
	3. Increasing patients’ accountability in clinical decision making
	4. Patients’ treatment follow-up
	5. Cost reduction
**B: Patient- related barriers in using PDAs**	1. Patients’ level of education and knowledge concerns
	2. Patients’ cultural problems
**C: Physician-related barriers in using PDAs**	1. Lack of resources
	2. Lack of an evaluation system for monitoring patients and physicians’ rights
	3. Lack of an appropriate role model among medical instructors

### Recognized benefits of using the PDA

This category included the five following codes: 1) Enhancing the psychological impact of treatment, 2) Adherence to principles of medical ethics, 3) Increasing patient accountability in clinical decision making, 4) Patients’ treatment follow-up, and 5) Cost reduction, which are explained below:

#### Enhancing the psychological impact of treatment

According to the participating physicians, patients become more familiar with different treatments of a disease and their advantages and disadvantages by using PDAs. They therefore have a more realistic point of view and become psychologically ready for the probable side effects of the treatment. Physicians even believed that this sharing could have a positive indirect effect on treatment results. They believed that although all the side effects would not necessarily happen to a patient but it is necessary for them to be aware of them.

“*If something happens to patients and they don’t have any information, it is harder for them to accept it” (Neuro)*

The participating patients thought likewise. They thought that using this tool would reduce their doubts in decision making. This would have a positive mental effect on them and at the same time, increase their knowledge and share in making a right decision that would lead to a better result in treatment. According to the patients, presenting this tool would increase their trust in physicians and could prevent multiple and unnecessary referrals.

However, some physicians believed that explaining all the possible side effects of treatment could raise patients’ anxiety and doubts in making decisions, and could even make the patients quit altogether.

“*Sometimes giving information worsens the situation. Consider this; it would cause patients to complain more, because they don't see all the aspects. Seeing the other side of the coin has its own problems.” (Endo)*

“Patients face a dilemma and it becomes difficult for them to decide. This negatively affects their decision making.” (Endo)

However, most physicians believed these doubts would not arise in all patients, but mostly in those with dependent and obsessed personalities.

#### Adherence to principles of medical ethics

According to the physicians, taking patients’ autonomy into consideration is one of the main principles of medical ethics, i.e. patients should have as much autonomy as possible. By using PDAs, patients’ beliefs and values will be respected. In turn, these values will be considered in their decisions. One of the physicians believed that:

“*By using this method patients are given beneficence which lets them have at least a primary knowledge of their disease and helps them realize where they stand.” (Onco)*

Valuing patients’ rights was one of the benefits that patients mentioned in this study.

#### Increasing patient accountability in clinical decision making

The participating physicians believed that patients have gotten used to not taking responsibility for decisions and prefer physicians to be the decision makers. Using this tool can make it possible for them to take part in decision making and to take some responsibility for it. In this regard some physicians said:

“*When making a decision it seems you are responsible for everything; but when you decide together with the patient both of you will be held accountable and not you alone* ”. *(Gen. Surg)*

According to the physicians, this tool gives accurate and evidence-based information to patients, and may prevent referring to unreliable databases and can prepare them to take part in decision making. The patients also confirmed this idea.

“*I like to choose. Choosing for one self is much better than letting the physician decide. When there is no other way the physician is asked to choose.*” *(W-LSES)*

“*Yes, unfortunately physicians neither explain such things to patients, nor expect such from them.*” *(M-USES)*

#### Patients’ treatment follow-up

According to the participating physicians, giving more knowledge to patients and involving them in decisions would make them more cooperative and engaged in their treatment. Henceforth, they would make regular visits to follow up their treatments.

#### Cost reduction

Both participant groups believed using PDAs would increase the frequency of a correct and appropriate decision, and may cut unnecessary costs which may arise because of wrong decisions, and eventually decrease total costs.

“*Eventually costs may become effective, because it prevents making inappropriate decisions.” (Uro)*

Some of the physicians also believed that the issue of cost reduction needs special cost-effectiveness studies and that such a conclusion could not be so easily drawn.

### Patient-related barriers in using PDAs

#### Patients’ level of education and knowledge concerns

In some physicians’ beliefs, differences in patients’ levels of education are among the main challenges in using PDAs. This is problematic especially when most of the patients are not highly educated. Some physicians thought that irrespective of the patients’ level of education, they had less information than physicians and this gave them less scientific power, thus they would face problems in deciding. But most physicians believed that even people of poor socioeconomic status have understood this tool, and essentially using this tool does not depend on the patients’ level of education.

“*Most of our patients and their families aren't much different from each other when it comes to decision making.*” *(Ophth)*

Although some physicians suggested categorizing PDAs according to patients’ education levels, most others disagreed with this suggestion. They thought PDAs could be designed in a scientific and simple way that suits any level of education.

In order to make PDAs more understandable, some physicians suggested using treatment algorithms but others thought this might be expensive and make PDAs suitable only for a specific group of people. As Iranian culture is more of an auditory culture and the rate of reading is low among most of the patients, according to some physicians, using educational movies as complimentary or replacement tools for handbooks could be really helpful.

“*Most of our patients like to simulate conditions for themselves and to compare themselves and their conditions with others.” (Ophth)*

Some patients suggested presenting existing information in educational CD formats as a replacement for handbooks. They also thought that illiterates could get help from their friends or relatives on how to read and understand PDAs.

#### Patients’ cultural problems

According to some of the participating physicians, patients are not used to being the decision makers. This habit can make them resist the use of PDAs.

“*Patients say: “whatever ‘your’ opinion is doctor, if you think that this (treatment) is going to work for me, then do it*”. *(Endo)*

According to some physicians, patients’ lack of familiarity with their own rights is another cultural challenge. Recommendations made by the physicians were that patients should be aware that making a decision and selecting the appropriate treatment requires them to have necessary and adequate information about the disease and its treatments. Therefore, we should increase the patients’ knowledge level in a way that they demand their rights from physicians.

“*Certain trainings are necessary for our patients; meaning they need to know that today’s medical methods are not like mathematical equations. Most of the times patients choose what should be done for them by considering its benefits and side-effects.” (Onco)*

Another problem stated by physicians was patients’ unfamiliarity with the principles of decision making which makes the process harder for them. One physician said:

“*Perhaps people outside Iran are more familiar with the language of science and uncertainty, so we should work more on this concept.” (Onco)*

Another solution suggested by physicians was to consider patient’s mental, spiritual and physical conditions at the time of presenting PDAs, to increase their cooperation. Therefore, patients should be in a mentally calm situation and have enough time for a mutual discussion with the physician. Presenting this tool at the time of patients’ admission would give them adequate time to decide.

According to the physicians, patient opinion has usually gained less direct attention. Therefore, asking for the patient’s opinion about decisions can be misunderstood as the physician not having enough knowledge and experience.

“*When you involve a patient, her/his impression is that “if the physician knew what s/he should do s/he would not have asked me”.*” *(Endo)*

Whereas patients believed that not only does involving patients in decision-making not cause a misunderstanding of the physicians’ knowledge, but that it would increase their trust and confidence in them too.

Patients believed that people should be acculturated to the use of this tool before its widespread application. Acculturation can be done through public media such as television, radio, newspapers, and magazines.

### Physician-related barriers in using PDAs

This category includes three codes, 1) Lack of resources 2) Lack of an evaluation system for monitoring patients’ rights in decision makings 3) Lack of an appropriate role model among medical instructors; as explained below:

#### Lack of resources

According to the participating physicians, lack of specialists per capita and consequently lack of time and increasing workloads have lessened physicians’ tendency toward using this tool and involving patients in decision making.

“*If the patient wants more information, it will take more of the physician’s time and this is not good for doctors.” (Endo)*

Some physicians suggested using expert teams or trained nurses to confront the problem of time shortage. However, in Iranian culture, replacing the physician with someone else in the medical process is not particularly favored.

“*Patients want to see their physicians, no matter how good and reliable I am as the physician’s assistant. Eventually the patient wants to see the physician.*” *(Neuro)*

Some physicians mentioned that the success in using trained experts depends on the type of disease and its treatment; meaning, experts cannot be used in complicated cases.

Patient opinion about trained nurses was that the nurse should be verified by the Ministry of Health or one of the specialists trusted by the patient.

Another problem mentioned by a number of physicians was the unrealistic tariffs. In some physicians’ opinions, unrealistic tariffs have caused physicians to sign contracts with specific pharmaceutical companies to compensate for low tariffs and consequently, leading patients to use those specific medications or treatments.

To confront this problem, physicians suggested setting rational tariffs for those physicians who use PDAs to make them more motivated in using them. In their opinion, there should be certain tariffs compensating the physicians’ time economically, otherwise their motivations for using the tool would decrease. One physician said:

“*The physician who spends enough time on the patient should not receive the same benefits as the one who doesn’t.*” *(Neuro)*

Besides, the physicians believed that using this tool would be eventually practical in some particular centers but that it would not have much applicability as a norm in clinics and hospitals in Iran, due to limitation of resources.

Patients participating in the research thought that physicians do not spend much time and effort on patients and the physician-patient consultation is limited to some short questions about the signs and history of the disease. Thus, this tool can compensate for this shortcoming and allow physicians to present information to patients and consult with them.

“*Unfortunately they don’t even spend 5 minutes with the patient. I had to refer to several physicians for this knee problem that I have.*” *(W-USES)*

Physicians and patients disagreed on the effect of this tool on physicians’ lack of time. Physicians believed using this tool required spending more time with patients. On the contrary, patients thought using this tool would save physicians’ time.

#### Lack of an evaluation system for monitoring patients and physicians’ rights in decision making

Physicians thought lack of an evaluation system for engaging patients in decision making would make physicians act according to their own preferences. In other countries like Canada where this tool is being used, evaluation systems are very systematic and principled. In one of the physicians’ opinion:

“*Why doesn’t evidence-based medicine work in our treatments? Because the physician who acts evidence-based isn't treated differently from a physician who does not! Acting non-academically makes more money and creates more motivation.*” (*Gastro*)

“*Medical ethics should be important to physicians and they should know that if they don’t adhere to them they will be considered as serious illegal acts.*” *(Ophth)*

Although some physicians believed that if using the tool would become mandatory, it would assure the usage of PDA, the majority believed mandatory plans do not work and that physicians’ tendency to use this tool should be raised by removing the barriers in that context.

#### Lack of an appropriate role model among medical instructors

According to the participating physicians the following barriers existed in medical education: absence of trainings in the field of patient management, involving patients in decision making, respecting their rights, and taking responsibility for educating them.

“*Our training process is such that when a patient visits an attending physician, the student asks for his/her medical history and brings him/her to me and I tell the patient what s/he should do and that’s it! The examination is over. ” (Endo)*

They believed our medical trainings gave the students the impression that it is the physician who must decide. Theoretical trainings are not sufficient; students should learn these principles in practice.

“*Well, actually this needs acculturation, meaning, both its knowledge and culture should be passed on. Only then it can be done.*” *(Derma)*

To acculturate the physicians suggested identifying opinion leader doctors and familiarizing them with the principles of evidence-based and shared decision making. This way they could promote the reception of this tool in other medical staff as well, hence raising its applicability. To achieve this goal, professors could play the key roles. It was also suggested to familiarize the Ministry of Health with the subject and highlight its significance, as it plays a key role in implementation of new rules.

“*Often there are certain people in every group and hospital who are the key players; if those people accept that a thing is useful then this acceptance can influence others as well.*” *(Neuro).*

## Discussion

Our findings propose that both patients and physicians welcome shared decision making. The main barriers in using this tool which were mentioned by both groups of participants were: patients’ cultural problems, awareness issues including lack of patients’ knowledge and education, lack of education in shared decision making for physicians, both theoretically and in practice. Moreover, the inappropriate distribution of human resources and lack of a strong monitoring system in the health system were other barriers mentioned by physicians in this study. To encourage medical staff and patients toward shared decision making, and to promote the use of PDAs, the barriers and solutions need to be identified at different levels of the health system.

Results from the present study and of course others show that familiarizing patients with their rights is an effective way of increasing their tendency toward shared decision making. Hence focusing on raising patients’ awareness with the aim of familiarizing them with the principles of decision making and with their own rights is one possible solution. Since primary health services have the most referrals from different socio-cultural classes, it has been suggested to hold these awareness-raising programs at different levels, including primary levels [19].

An increased likelihood of the patient’s misunderstanding of the physician’s level of knowledge; an increased doubt in decision making, disagreements among physicians and patients’ choices, and lack of time were the barriers and challenges stated by physicians, about which the patients had completely different opinions. Physicians’ unfamiliarity with the principles of medical ethics e.g. patients’ rights and shared decisions- was mentioned as the underlying cause of these disagreements in another study [20]. Results from different studies show that moving toward greater patient involvement requires physicians to become more familiar with different concepts of decision making, patients’ preferences and kinds of decisions [20-22]. It is also recommended to pay more attention to evidence-based medicine, with an emphasis on values and principles of patient participation in decision making, as one of the main elements of medical students’ educational curriculum. Physicians’ lack of time was one of the barriers mentioned by physicians in our study. The high numbers of referrals made to public clinics and the inappropriate distribution of human resources were the main reasons behind physicians’ lack of time for involving patients in decision making. A great share of health services are being delivered by public clinics. That is why the number of specialists per patients referring to these centers should gain special attention. Since most public hospitals are in charge of training physicians, more human resources and costs should be allocated [19,23,24].

Participating physicians thought the cost of involving patients in decision making would not appear beneficial to physicians, because the time they spend on examining patients is not proportionate to their incomes. Not only can this problem marginalize patients, but it can also lead physicians into prescribing drugs from particular pharmaceutical companies or into advising specific treatments that have financial benefits for themselves. Results from Davis et al’s study also confirm these findings [25,26].

Although both physicians and patients recommended using a nurse or a trained expert to compensate for physicians’ lack of time, but according to McKeown patients and physicians differ in their perceptions of nurses as consultants, hence this can lead to confusion and misunderstanding in the patient-physician relationship [27]. Moreover, this solution can impose extra costs on the system for educating nurses. Another solution proposed by one of the physicians was to use educational videos. This intervention was believed to help raise patients’ perception of PDAs as well. Holmes’ study showed that although these systems could work as facilitators in shared decision making, but they decreased face to face and verbal relations between physicians and patients and could weaken the idea of consultation in decisions [26].

Improving skills for communicating with patients is a pre-requisite in shared and evidence-based decision making. This matter has been confirmed by other studies as well [19]. Hence, in addition to the theoretical trainings that medical students receive in the field of ethics and clinical decision making, it is strongly advised to practically strengthen their communication skills for building better relationships with patients as well.

Identifying influential people in hospitals was another solution underscored in this and other studies [28,29]. Influential persons are those individuals who can actually influence clinical performance in a specific setting [30]. Therefore, by using this tool and respecting patients’ rights in practice, academic professors can be suitable role models for students.

Creating incentives and evaluation systems for those who use PDAs and share clinical decisions with patients is another strategy that could be considered in this regard.

The findings of this study comply with other studies that suggest sharing decisions with patients in accordance with their culture, level of education, age and ethnicity [10,31]. Physicians should be flexible, and be able to adjust themselves to the type and amount of information correspondent to the patients’ education and requirement levels [19]. Furthermore, preparing culturally-appropriate PDAs in different fields could be another option in universities.

Likewise, according to the participating physicians and results of other studies, patients’ tendency toward getting involved in decision making depends on the type and severity of their disease [19,32,33].

### Study limitations and strengths

The qualitative nature of the study and the purposeful sampling done in it lowers the generalizability of the findings. Hence, interpretation of findings should be done with caution. As a further step, we recommend evaluating physicians’ opinions through questionnaires designed on the basis of the current study’s findings, with a larger and more generalizable sample size. However, maximum variation sampling was done for patients and physicians. And in spite of the fact that the tool had not been used in the country before, after conducting the interviews, we realized that the responses given by various specialties and patients from different socio-economic classes did not differ significantly. Even then, we must consider that saturation may not have been achieved from various layers of participants and we cannot be certain of the kind of saturation achieved. Nonetheless, to our best knowledge, no such tool has been used in low- and middle-income countries so far. The results of this study can therefore give an overview and prompt the usage of patient decision aids in developing countries such as Iran.

## Conclusions

In conclusion, it seems that the PDA tool has been accepted by the majority of Iranian patients and physicians under study. The applicability of this tool can be increased by laying greater emphasis on patients’ rights and medical ethics during the medical course, and informing patients of their rights. Therefore, it seems that planning can be done to design evidence-based PDAs that are based on patients’ culture and level of knowledge. Nevertheless, evaluating the cost-effectiveness of such tools through clinical trials seems necessary.

## Competing interests

The authors declare they have no competing interests.

## Authors’ contributions

HR was involved in the data collection, analysis and drafting of the manuscript. SN contributed to the idea and study design. She actively took part in the revision of the manuscript. RM was involved in the study design, and drafting of the manuscript. JG and BS participated in the study design and data analysis. LH participated in the data collection and analysis. FD and AR were involved in the study design. All the authors read and approved the final manuscript.
